# Sensory Polymers: Trends, Challenges, and Prospects Ahead

**DOI:** 10.3390/s24123852

**Published:** 2024-06-14

**Authors:** Cintia Virumbrales, Raquel Hernández-Ruiz, Miriam Trigo-López, Saúl Vallejos, José M. García

**Affiliations:** Departamento de Química, Facultad de Ciencias, Universidad de Burgos, 09001 Burgos, Spain; mtrigo@ubu.es (M.T.-L.); svallejos@ubu.es (S.V.); jmiguel@ubu.es (J.M.G.)

**Keywords:** sensory polymers, polymer sensors, chemosensory polymers, polymer chemosensors, chemical sensors, chemosensors, frontier in sensor technology

## Abstract

In recent years, sensory polymers have evolved significantly, emerging as versatile and cost-effective materials valued for their flexibility and lightweight nature. These polymers have transformed into sophisticated, active systems capable of precise detection and interaction, driving innovation across various domains, including smart materials, biomedical diagnostics, environmental monitoring, and industrial safety. Their unique responsiveness to specific stimuli has sparked considerable interest and exploration in numerous applications. However, along with these advancements, notable challenges need to be addressed. Issues such as wearable technology integration, biocompatibility, selectivity and sensitivity enhancement, stability and reliability improvement, signal processing optimization, IoT integration, and data analysis pose significant hurdles. When considered collectively, these challenges present formidable barriers to the commercial viability of sensory polymer-based technologies. Addressing these challenges requires a multifaceted approach encompassing technological innovation, regulatory compliance, market analysis, and commercialization strategies. Successfully navigating these complexities is essential for unlocking the full potential of sensory polymers and ensuring their widespread adoption and impact across industries, while also providing guidance to the scientific community to focus their research on the challenges of polymeric sensors and to understand the future prospects where research efforts need to be directed.

## 1. Introduction

Sensory polymers have gained recognition in the field of sensor technology, given their ability to react to changes in their environment in a specific, reproducible, and usable way, aiming to mimic and even go beyond natural sense organs. Smart polymeric materials with sensory capabilities can modify their physical and/or chemical properties under the influence of external stimuli (e.g., the presence of chemical species and bioactive molecules, pH, temperature, mechanical forces, light radiation, magnetic fields, electric fields, etc.), providing information about their environment. Thanks to the huge variety of their tuneable physical and chemical attributes, polymers have proved to be of great use in the development of sensory devices and can adapt to many applications.

From early low-molecular-weight chemosensors to more sophisticated sensory polymers, this field of study has experienced exponential growth in the number of publications, active researchers, and financial investment in recent years [1]. Their transformative role in detection technology has led to numerous advancements. However, the field faces several ongoing challenges that demand relentless scientific investigation and innovation. For this reason, this review aims to comprehensively analyse the latest achieved progress, exploring upcoming trends encompassed by sensory polymer technology and identifying the challenges that must be overcome to fully exploit the variety of potential outcomes they are intended to achieve.

Firstly, there exists an unending requirement to engineer novel chemosensors with the capability of detecting analytes that are still unknown. This extends beyond creating sensors for emerging biomarkers or detecting trace contaminants in our environmental surroundings, encompassing air and water supplies. Moreover, the standards set by regulatory authorities are becoming increasingly stringent in the realm of biological and environmental control analyses. Consequently, although currently existing chemosensors may demonstrate functionality, they often lack the selectivity and sensitivity for specific real-world applications. Tailor-designed custom receptors for unique targets and the improvement of existing sensor systems are essential, thus the demand for sensors persists as challenges continue to arise [2].

On the other hand, polymers provide numerous benefits for sensor technology, surpassing traditional chemosensors in various ways. Their advantages include being lightweight, flexible, and biocompatible, as well as offering easy functionalization and efficient, low-cost production methods. Polymers can directly participate in detection reactions or serve as platforms for receptor attachment. They are adaptable for use on diverse substrates and can be tailored to create user-friendly sensory devices. Furthermore, modifying the chemical structure of polymers can enhance properties like selectivity and response time for future sensor systems [3,4].

**Figure 1:**
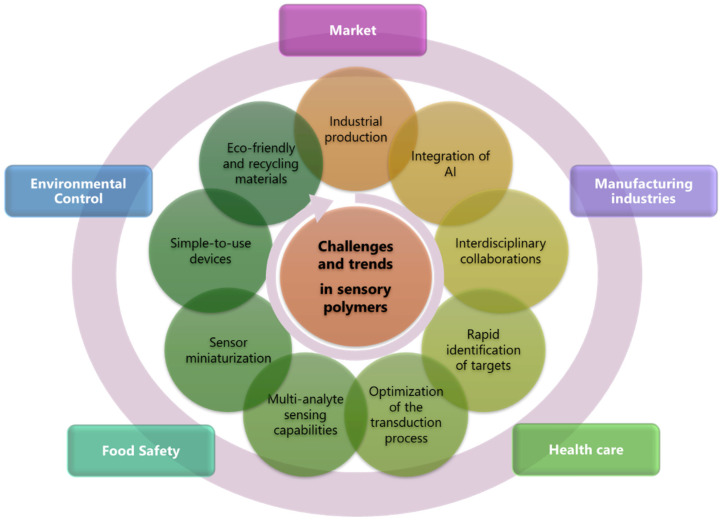
Recent challenges and trends in sensory polymers.

Despite efforts to improve current sensors, their market presence has only seen slight improvements (Figure 1). A collaborative approach involving chemistry, physics, engineering, and data and computer sciences is crucial to address these challenges and achieve large-scale progress and manufacturing of these technologies. 

In this scenario, polymeric sensory materials are expected to play a significant role in various domains, such as medical applications, chemical reaction control, and gas identification, and they can potentially serve as electronic noses or tongues [5,6,7]. Researchers in the field stand on the threshold of pioneering advancements, poised to overcome remaining challenges and fully harness the potential of sensory polymers in real-world applications, extending their use beyond the laboratory and into everyday life [8].

Given the need to address emerging analytical requirements, comply with regulatory guidelines, and continuously improve chemosensor performance to meet the evolving demands of practical applications, this review will explore the next frontier in sensor technology, where polymers are not just passive detectors but active participants in data collection and environmental interaction. The discussion will cover anticipated innovations in materials science, potential applications in various industries, and the expected integration of sensory polymers with other technological advancements such as artificial intelligence (AI) and the internet of things (IoT). Additionally, emphasis will be placed on the challenges facing the commercialization of these technologies, highlighting the need for scalability, reproducibility, and practical integration.

In the interest of enhancing readability and comprehension for the reader, our goal is to condense approaches and consolidate criteria to create a unified classification of polymeric sensor materials, meticulously selecting both the polymer family they belong to and the type of sensor, considering different targets as well as the various application fields where there is currently more significant innovation, and more challenges are expected in the future. In this review, firstly, we will take a forward-looking view of different types of polymeric materials used in sensor applications, examining their physical/chemical structure, composition, inherent properties, typical characteristics, and how they will change across short-, medium-, and long-term horizons in various domains. Secondly, we will focus on specific types of polymeric sensors designed for the detection of particular targets. For instance, these targets may include physical parameters, such as temperature, pH, humidity, and chemical compounds, or biological substances ranging from drugs and proteins to pathogens. Additionally, we will define the devices in which these polymeric sensors are employed, along with their respective challenges. We will also encompass the current challenges and trends facing these polymeric materials and their integration into the market and engagement with society. 

## 2. Types of Polymeric Materials for Sensing Applications

In terms of materials, our discussion will primarily focus on molecularly imprinted polymers (MIPs), conducting polymers (CPs), nanocomposite polymers (NCPs), hydrogels, and acrylic polymers. These materials are chosen due to their widespread use in sensor technology and their current advancements, making them the subject of ongoing research and improvement.

### 2.1. Molecularly Imprinted Polymers (MIPs)

MIPs are synthesized using template polymerization techniques to create artificial recognition systems with specific binding sites or recognition cavities for target molecules. This technology offers a synthetic route to develop highly selective polymers that mimic natural receptors, providing precise molecular recognition essential for sensor applications. MIPs overcome the limitations of biological recognition elements and can selectively bind target molecules based on their shape, size, and functional groups. Their durability, reusability, and tailored specificity render them crucial tools for achieving accurate detection across various environments. They can be designed to identify a broad spectrum of substances, ranging from proteins, enzymes, bacteria, viruses, and toxins to metals and ions [9,10] These attributes position MIPs as highly versatile and cost-effective alternatives to conventional detection methods [11]. Moreover, ongoing advancements in MIP technology continue to enhance their capabilities, further expanding their potential applications [12].

**Figure 2:**
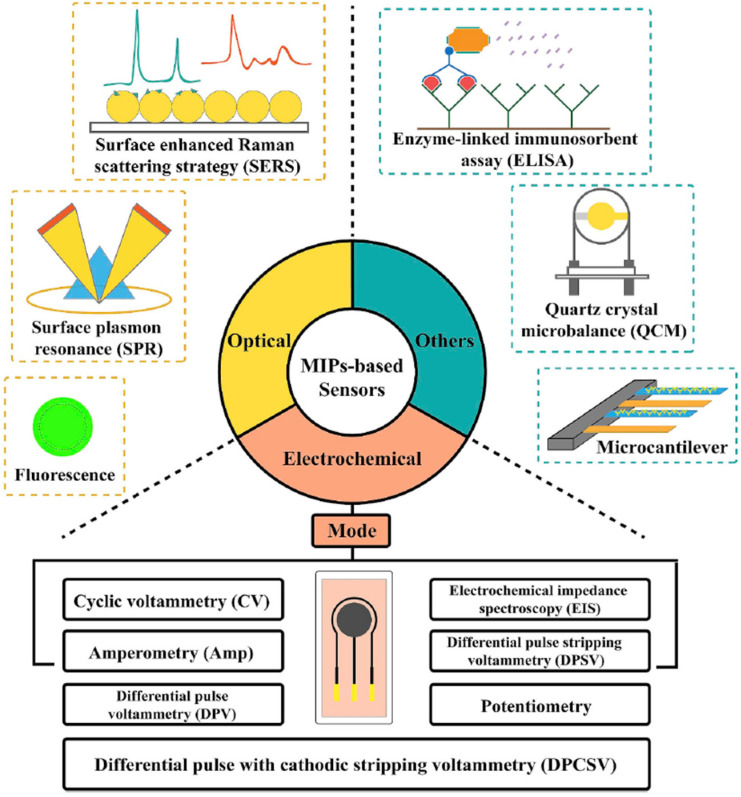
Schematic representation of various types of MIP-based sensors (reprinted from [12], Copyright (2024), with permission from Elsevier).

There is a wealth of information related to electrochemical and optical sensing, both commonly utilized analytical techniques combined with MIPs, demonstrating their versatility and broad design possibilities. MIPs can exhibit various types of responses, including optical responses such as surface-enhanced Raman scattering (SERS), surface plasmon resonance (SPR), or fluorescence upon binding with target molecules. They can also demonstrate electrochemical responses or be integrated into sensing platforms like ELISA (enzyme-linked immunosorbent assay), microcantilevers, or quartz crystal microbalances, providing versatile detection capabilities for specific analytes. Figure 2 summarizes various MIP-based sensors and their working principles. However, this review aims to not only discuss the advancements but also to highlight the challenges associated with MIPs when they are integrated into sensor devices. 

In this sense, MIP technology challenges persist, such as restricted dynamic range and potential cross-reactivity in complex matrices. Future advancements in MIPs are aimed at enhancing both selectivity and sensitivity, broadening the range of detectable substances, and leveraging nanotechnology to achieve superior sensing capabilities. For instance, in antibody-related diagnostic sectors, valued at USD 43 billion in 2021, MIPs have the potential to rival antibodies by providing cost-effective assay development and streamlined reagent preparation, achieving significant figures in this market [13,14].

In this context, MIP-based plasmonic sensors are currently highly regarded and attractive, especially in the biomolecular detection area [15]. These materials combine MIPs with plasmonic technology to detect and quantify the presence of specific analytes in a sample. These polymers leverage plasmonic properties such as surface plasmon resonance (SPR) or localized surface plasmon resonance (LSPR) to generate optical signals that vary in response to changes in the concentration of the target analyte. The combination of SPR or LSPR technology with MIPs offers advantages such as real-time analysis capability and label-free operation, high selectivity and sensitivity, and compatibility with complex matrices [16]. They can be compatible with a wide range of targets, including toxins, biomarkers, or environmental contaminants, finding applications in fields like medicine, food safety, and environmental monitoring.

However, they also face challenges similar to other MIPs, such as the optimization of selectivity and sensitivity, ensuring long-term stability, and achieving miniaturization for portable applications [17].

The integration of MIPs with nanotechnology presents novel opportunities, including, for instance, the incorporation of carbon dots (CDs) and metal nanoparticles. Ever since their inception in 2004, CDs have attracted considerable interest due to their physicochemical properties, optical characteristics, biocompatibility, and environmental sustainability, positioning them as promising candidates for fluorescent probes in nanosensor applications [18].

**Figure 3:**
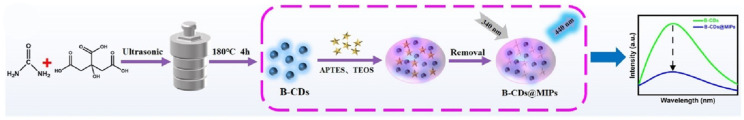
Representation of green synthesis process of CDs/MIP (reprinted from [20], Copyright (2024), with permission from Elsevier).

On the one hand, the fusion of MIPs with green CDs addresses current challenges, notably the necessity for enhanced selectivity and sensitivity towards specific substances, enabling precise and selective detection in complex matrices. Leveraging the robustness and reusability of MIPs alongside the advantageous attributes of CDs, such as water solubility and photostability, positions MIP/CD-based sensors as potent tools across diverse applications in different fields, promising significant strides in biology, food safety, and environmental surveillance. By embracing green chemistry principles for reusable and environmentally conscious sensing solutions and innovating imprinting techniques for micro- and macromolecules, the scope of these sensors in detecting viruses, bacteria, and proteins will further expand [19,20]. It is worth mentioning that lifetime detection methodologies and ratiometric sensing are augmenting the probing capabilities of MIPs/CDs (Figure 3).

On the other hand, the synergy between metal/metal oxide nanoparticles (NPs) and MIPs is propelling sensor technology forward, providing optical, catalytic, and conductive properties to enhance sensor performance. This advancement facilitates the development of enzyme-free biosensors and more effective electrochemical reactions. Although selectivity and stability need additional improvement, the possibilities for these composites in sensor advancement are extensive, progressing toward new applications and eco-friendly synthesis methods [21]. Future research includes decreasing non-specific adsorption to enhance MIP selectivity, investigating 2D materials such as MXene (material composed of transition metals, carbon, and/or nitrogen, with unique properties) for increased sensitivity, and designing MIPs for biological entities to boost disease biomarker detection [22].

Regarding molecularly imprinted fluorescent sensors (MIFs), these combine the high selectivity of MIPs with the sensitive response of fluorescent materials, converting molecular recognition into readable fluorescence signals. MIFs enhance the performance of molecular imprinting and broaden its applications, facilitating high-efficiency enrichment and sensitive detection of trace substances in complex matrices. Compared to traditional analytical techniques, MIFs offer high sensitivity and selectivity, showing significant potential for rapid detection of targets in food and environmental safety. Despite their potential, innovative MIFs face challenges, such as the development of new synthesis methods, given the disadvantages of existing ones, such as irregular shape, heterogeneous particle size, and prolonged polymerization time. Additionally, understanding the molecular imprinting process, recognition mechanism, mass transfer, and characterization of polymer structure requires further research. Improving selectivity, mass transfer rate, and adsorption capacity, alongside developing specific functional monomers for template molecules, are necessary. The greatest challenge of these polymers lies in multitasking detection in food matrices, requiring MIFs capable of simultaneously detecting multiple factors [23].

### 2.2. Conducting Polymers (CPs)

CPs feature extended π-conjugated systems that enable the movement of electrons along their chain. This characteristic gives rise to unique electronic and optical properties, allowing these polymers to conduct electricity to a limited extent, like semiconductors. They can exhibit responsive behaviour to stimuli like electrical fields or chemical interactions, making them useful for diverse sensor applications where changes in electrical conductivity or optical properties are crucial for detection and analysis. They represent a unique class of polymers that have attracted significant attention in the development of chemical- and bio-detection strategies due to their high electrical conductivity, low cost, and good processability. These qualities position them at the forefront of industrial needs.

The latest research endeavours address the challenges of achieving greater efficiency and autonomy, surpassing traditional materials in efficiency and compatibility with computing analysis, and also functioning with minimal human supervision. Moreover, challenges such as enhancing stability, reproducibility, and device integration are being tackled to improve the practicality of CP sensors. This is why three research trends are emerging in the field of CPs, focusing on miniaturization, process automation, and multitasking devices [17].

Most CP backbone chains consist of alternating single and double bonds based on either polyenes or aromatic rings, which grant a conjugated structure. The most relevant CPs in sensor technologies are polypyrrole (PPy), polyaniline (PANI), and polythiophene (PTh), due to their distinctive alterations in electrical conductivity triggered by environmental stimuli. These polymers facilitate the development of exceptionally sensitive and selective sensors suitable for various applications, ranging from gas detection to biomedical diagnostics [24,25], which will be further described in the corresponding section. For example, CP-based gas sensors can detect low concentrations of harmful gases by exhibiting significant changes in resistance or capacitance upon exposure, making them invaluable for air quality monitoring and industrial safety [26,27,28]. In the biomedical field, CPs are integrated into biosensors by coupling with biological recognition elements to detect disease biomarkers with high specificity. 

Prospective investigations aim to customize CPs through chemical modifications or integration with nanotechnology to achieve superior performance, facilitating their incorporation into portable, flexible, and wearable devices. For instance, the synergistic effect between CPs and carbon nanotubes (CTs) results in the material’s enhanced chemical and electrical properties. These CP/CT nanocomposites, utilizing polymers like PANI, PPy, PTh, and poly(o-phenylenediamine) (oPD), have been effectively employed for sensor applications to detect a wide range of biomolecules, gases, metal ions, and environmental pollutants. Additionally, this type of nanocomposites is highly versatile and is also employed in strain and pressure sensors. However, further research is required to improve their selectivity, sensitivity, response times, detection limits, and recovery, enabling more effective and innovative technologies and broader applications [29].

### 2.3. Polymer Composites (PCs) and Nanocomposites (PNCs)

In the past few years, composites have become one of the most promising substitutes for traditional materials, offering an anticipated set of augmented physiochemical properties designed for specific applications. Polymer composite sensors are a class of sensory materials where polymer matrices are blended with one or more filler materials (e.g., glass fibres, talc, silica, carbonaceous fillers, etc.), commonly known as reinforcements or additives. These fillers, which can take the form of particles, clays, fibres, microspheres, or other components, are integrated into the polymer matrix to deliver improved sensor characteristics, rendering it, for instance, more stable, sensitive, selective, or reactive to stimuli in a cost-effective manner.

As an example, it is possible to design colourimetric mechanical sensors resulting from the combination of polymer matrices with mechanochromic materials [30]. These advanced materials have the peculiarity of converting mechanical stimuli (e.g., strain, force, pressure, and impact) into observable changes in colour. The advantage offered by these materials is twofold: the durability and reusability of the sensors are ensured by the polymer matrices, whereas the mechanochromic components provide a distinctive colour response to different types of mechanical stimuli. As research advances, the potential of these sensors spans a wide range of applications within the construction safety and transportation sectors, as they enable rapid and equipment-free detection of mechanical stress or failure.

**Figure 4:**
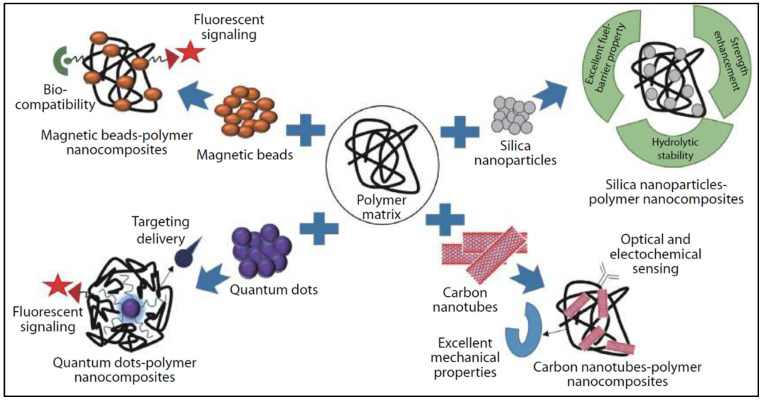
The selection of filler nanomaterials for the target properties. The choice of a particular nanofiller exerts a considerable impact on the properties of the polymer nanocomposites (Reprinted from [31], Copyright (2024), with permission from Springer Nature).

Due to the extensive research performed in the field of nanotechnology in the past few decades, PNCs have attracted much attention. They are formed by combining polymers and metal/inorganic nanofillers leading to new functionalities and extraordinary performance (Figure 4). The synergy between the polymeric matrix and the dispersed nanoscale fillers enhances their mechanical, optical, and electrical properties on the microscopic scale, which has raised considerable interest due to their emerging applicative potential in various scenarios. The combination of such enhanced properties along with the possibility to be manufactured in a variety of forms (films, fibres, or coatings), make them ideal for the development of chemical and biological sensors, offering significant advantages such as substantial electrical conductivity, high mechanical sturdiness, lightweight, and increased chemical resistance, sensitivity, selectivity, and response times.

Nanoscale fillers can range from unidimensional nanowires and nanotubes, bidimensional graphene and nanoclays, to 3D cubical and spherical nanoparticles. As for microscopic fillers, different materials can be employed, including the above-stated traditional materials, metals, metal oxides, and carbon derivatives. The nature of such nanoscale fillers and the selected polymeric matrix drastically impacts the physiochemical behaviour and sensing potential of PNCs. For example, graphene and other carbon-based materials, known for their remarkable electrical and mechanical properties as well as extensive surface area, have garnered considerable research interest, particularly when combined with CPs like PANI, PPy, and others, to generate novel conducting nanocomposite materials that can be employed as gas chemiresistive sensors of applicability in both fundamental research and industry. These composites surpass the individual properties of each isolated material, providing improved gas-sensing capabilities, even under ambient conditions [32].

Nanostructured polymers significantly influence biological and technological fields, particularly in drug delivery, catalysis, and sensing applications. Recent breakthroughs in nanotechnology/wearable devices have emerged as significant progress in health care and medical diagnostics, robotic systems, prostheses, visual realities, and professional sports. These materials, encompassing capacitive, piezoresistive, and piezoelectric varieties, are garnering interest due to their heightened sensitivity, cost-effectiveness in production, and compatibility with biological systems. In the field of mechanical sensing, the trend in sensor design towards material and functional diversification marks a notable advancement, highlighting the growing importance of visual diagnostics in real-world applications [30].

Despite the discussions about their formation mechanisms, the investigation of PNCs remains a crucial focal point, requiring extensive scientific effort to fully harness their capabilities and understand the fundamental principles dictating their functionalities, along with the design of biopolymer nanocomposites presenting a non-toxic, biodegradable, and biocompatible option [31]. 

### 2.4. Polymer Hydrogels

Hydrogels are an important class of polymeric materials widely used for developing functional interfaces and sensing platforms due to their excellent intrinsic properties, including sensitivity to external stimuli and tuneable mechanical properties. They are highly hydrophilic three-dimensional polymeric materials capable of retaining large amounts of water (>90%). Hydrogels present a crosslinked structure formed by either covalent or non-covalent interactions (i.e., electrostatic, hydrophobic, coordinative, dipole-dipole, or chain entanglements), and their water affinity is due to the presence of hydrophilic functional groups like -OH, -CONH_2_, -NH_2_, -COOH, -SO_3_H, and -CONH-. Although they are not soluble in water, they can absorb and retain large amounts of either water or biological fluids, making them suitable for sensing applications involving changes in hydration or swelling behaviour in response to specific stimuli. In addition, their water-uptake capability provides an appropriate environment for biomolecules, facilitating their long-term bioactivity and being particularly useful in biosensing [33]. Their tuneable mechanical properties and biocompatibility render them ideal for applications in tissue engineering, wound dressings, drug delivery, biosensing, and environmental monitoring, providing early insights into infection or contamination [34].

Hydrogels can undergo volume–phase transitions when exposed to specific stimuli or after interactions with the analyte, leading to a change in properties such as swelling, collapse, transparency, solution-to-gel transition, and conductivity, which can be exploited for the construction of sensor devices. The detection methods used in hydrogel sensors can be divided into two main categories. On the one hand, optical techniques rely on a variation of an optical property upon interaction with the analyte, including colour, absorbance, fluorescence, or volume. Alternatively, polymer gels can be used as electrochemical (bio)sensors, achieving conducting properties by doping with conductive materials (e.g., CPs, carbon materials, metal nanoparticles, or quantum dots). The resulting electroconductive hydrogels are responsible for (bio)recognition of the target, which triggers a change in the electrochemical signal recorded as current, voltage, or impedance.

**Figure 5:**
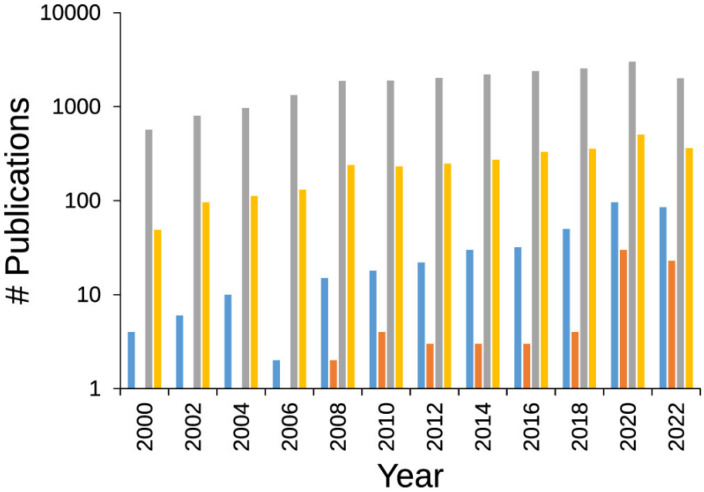
Number (#) of publications (semi-logarithmic axes) in scientific databases obtained after searching for “conducting polymer hydrogel” (in blue), “conducting polymer hydrogel sensor” (in orange), “conducting polymer” (in grey), and “conducting polymer sensor” (in yellow) keywords (reprinted from [1] under CC BY 4.0 license).

The growing interest in this type of materials is evident in the evolution of ongoing scientific studies with conducting polymer hydrogels (CPHs), which has significantly increased over the past decade. A thorough examination of the literature using specific keywords to differentiate between CPs and CPHs, and their application to sensors, reveals an intriguing publication trend (Figure 5). Furthermore, more specialized scientific studies focusing on the use of CPHs as sensors have experienced a substantial increase in the last two or three years, with the number of papers published multiplying by six compared to those published in the 2018–2019 biennium. This sudden shift in trend underscores the importance and interest that this type of material has garnered for its specialized application in sensor probes.

In recent years, hydrogels have positioned themselves as the leading biomaterials in the medical field, and remarkable progress has been observed in the utilization of hydrogels and CPHs for diagnostic, wearable, and implantable biomedical sensors. The facile modulation of their properties grants the creation of tailor-made and the fine-tuning of the material functionalities, allowing the fabrication of refined sensors that meet the market needs. For instance, the flexibility and resilience attributes of this type of polymer have positioned them at the forefront of innovations in human health monitoring, such as electronic skin (e-skin) sensors. CPHs are particularly well-suited for these applications due to their inherent high mechanical strength and stretchability, essential qualities for wearables requiring durability and flexibility. 

Based on the most recent work conducted in the field of hydrogels, there lies tremendous potential for future scope. CPHs exhibit numerous superior characteristics compared to conventional hydrogels and other materials, including enhanced electrical and mechanical properties, alongside electronic and ionic conductivity. Their self-healing capability is crucial, but establishing their specific suitability for biomedical sensor applications remains lacking. Additionally, their excellent adhesion and biocompatibility make them ideal for tension-detection devices in human health monitoring. Unlike traditional hydrogels, they are easily sterilizable; however, achieving fully bioabsorbable portable biosensors remains a challenge due to the required electronics. Common methods for CPH preparation include doping, mixing, and copolymerization, while 3D printing is considered an advanced technique. The use of MXenes and the design of electronic skin devices are also promising strategies. However, advanced CPHs with specific characteristics and highly sensitive sensors are needed for practical applications in human health care.

Minimizing the complexity of hydrogel formulations and increasing their durability, adaptability, and biodegradability are some of the primary priorities for the upcoming future. Moreover, there is promise for greener and eco-friendly solvent-less synthetic methods of grafting and crosslinking of hydrogels, and novel biomimetic materials with reduced toxicity are being explored for drug delivery devices, tissue and molecular engineering, implants, and gene therapy. Furthermore, in vitro assays often prove insufficient to justify advancement to animal trials. Although animal models present various challenges, such as ethical concerns, limited translatability to humans, low efficiency, and associated time and financial costs, it remains essential to examine the effects of CPHs across multiple organs and in whole organisms. This is particularly true for CPHs used in implantable sensors.

### 2.5. Acrylic Polymers

Acrylic polymers serve as versatile sensing materials in specialized polymer sensor applications. These polymers offer tuneable properties such as swelling behaviour, mechanical strength, and chemical stability by carefully selecting the nature of the constituting monomers, making them ideal for sensing environmental changes. The commonly used acrylic polymers are derivatives of acrylamide, their copolymers, and meth/acrylic acid. Acrylic-based sensors can be tailored by incorporating responsive components and thus they have gained prominence in the sensor technology research field, fundamentally due to their capacity for easy modification of their internal chemical structure. This adaptability of acrylic polymers, achieved by incorporating a broad spectrum of sensing groups, enables the detection and identification of various analytes or stimuli, which explains their widespread use in sensing applications. 

The selection of sensing groups plays a crucial role, as it determines the sensitivity and selectivity of the material towards specific stimuli. A diverse range of monomers can be copolymerized to incorporate the intended functionality into the acrylic material. For example, incorporating ionic groups into acrylic polymers can make the material sensitive to electrolyte concentration or changes in pH, rendering them ideal for ion-selective electrodes or pH sensors. Another option is to introduce hydrophobic groups into the polymeric matrix to develop sensors capable of detecting organic or non-polar compounds. The physical structure of the sensor, whether it be a thin film, a coating, a fibre, or a bead, already presents a challenge. On the one hand, it must carefully align both the properties of the polymer and the requirements of the application, along with consideration of factors such as the operating environment, the durability of the sensor’s response, and the possibility of interference from external substances. On the other hand, the area and sector of application must also be considered, encompassing applications for environmental monitoring, food safety, and medical diagnostics, among others [35,36].

Recently, solid-state chemistry has enabled the development of materials containing chemically unstable groups even under ambient conditions, taking advantage of the different chemical behaviours exhibited by macromolecular environments compared to low-molecular-mass entities [37].

## 3. Targeted Sensors

The remarkable advancements in polymer sensor technology have prompted the creation of inventive methodologies to address contemporary challenges. Progress in polymer chemistry has streamlined the production of new materials endowed with heightened sensitivity and selectivity tailored for distinct stimuli. Correspondingly, a range of sensor types has been devised, each attuned to a particular stimulus. This section endeavours to delineate the extensive array of sensors, which mirrors the diversity of stimuli or analytes they are engineered to discern and characterize. Furthermore, illustrative examples of real-time applications are provided within the framework of the described polymer-based sensor types.

Among their endless uses, polymeric sensors are set to enhance food safety and quality control by integrating smart packaging to detect contaminants—including biological and chemical substances, allergens, nutritional ingredients, and potentially harmful food additives—alongside sensors monitoring temperature, humidity, gas levels, and pH to ensure the freshness and safety of food products [38,39]. Biomarker detection has emerged as a crucial approach for non-invasive health monitoring, offering comprehensive insights into both physiological and pathological health conditions. Meanwhile, the identification of environmental pollutants has become a significant focus, given their adverse effects on ecosystems, global sustainability, and human health. Contaminants such as heavy metals, pesticides, and volatile organic compounds (VOCs), among others, present risks that can lead to chronic illnesses, ecological deterioration, and toxicity. Consequently, the following description presents recent developments regarding challenges and trends in various types of sensory polymers aimed at addressing current concerns arising from the multitude of stimuli and analytes requiring detection.

### 3.1. Gas Sensors

The emission of gaseous pollutants such as NO_2_, NH_3_, CO, CO_2_, H_2_S, SO_2_, and other toxic gases from industry has become a major environmental issue that seriously threatens human health. The precise detection of such targets is vital to implementing effective environmental control strategies, improving air quality, ensuring access to drinking water, and meeting regulatory standards aimed at protecting both the environment and public health. In this context, gas sensors offer a promising and economical solution to problems related to hazardous gases and have the potential to transform our ability to monitor and respond to their presence, helping decision-making and leading to informed actions toward pollution mitigation and the promotion of a healthier planet. Moreover, the demand for reliable, cheap, tiny, low-power-consumption gas-sensing devices is currently quite substantial, not only for environmental monitoring but also for medical diagnostics and food quality control. From this perspective, gas sensors can contribute to the simple monitoring of the state of human health. In addition, they can help track changes in gas concentration in food packaging to detect food spoilage and microbial activity, thereby facilitating intelligent systems for predicting shelf life, monitoring nutrients, and securing comprehensive quality assurance throughout the food supply chain.

In this context, two main types of polymeric gas sensor materials exist. On the one hand, CPs (e.g., polyamides (PAs), PANI, PPy, PTh, etc.) and their composites with other polymers, such as polyvinyl chloride (PVC) and polymethyl methacrylate (PMMA), have undergone rapid development and shown promising applications due to their ability to modify electrical conductivity or resistance at room temperature when exposed to gas molecules with acid/base or reducing/oxidizing properties. However, due to their relatively low conductivity and high affinity for volatile organic compounds (VOCs) and moisture, they generally display low sensitivity, poor gas selectivity, and stability, which hamper their practical gas sensor applications.

**Figure 6:**
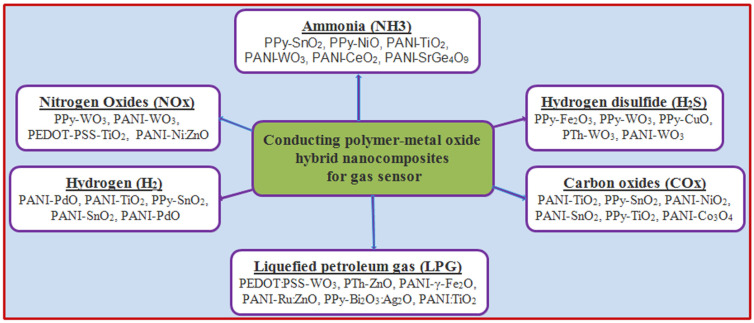
Gas-sensing applications of various conducting polymer–metal oxide hybrid nanocomposites (reprinted from [40], Copyright (2024), with permission from Elsevier).

On the other hand, hybrid CP–metal oxide nanocomposites have been developed in recent years as reliable and novel gas-sensitive materials that bring together the versatility and low working temperatures of CPs and the high sensitivity, fast response and recovery time, and large surface area of metal oxides (also widely used as single-component gas sensors). These materials can be easily tailored by varying their precursors. For instance, some commonly studied combinations have shown remarkably promising results, including PANI-ZnO, PPy-SnO_2_, PTh-TiO_2_, and poly(3,4-ethylenedioxythiophene) (PEDOT)-CuO, which display high sensitivity, selectivity, and stability towards various gases such as NO_2_, CO, NH_3_, and H_2_S (Figure 6). However, the performance of these sensors can vary depending on the synthetic methodology, the type of polymer, and nanoparticle size and concentration, which underscores the urgent need to conceptualize their underlying characteristics and optimize their structure. Particularly, an increase in surface area and number of active sites is sought to achieve a better sensitivity, which can be attained by selecting proper catalysts with high catalytic efficiency.

Therefore, there are still many challenges to be solved in the future in terms of enhancing the sensing capabilities and stability (especially against water) of current gas sensors to be able to function in a real-world environment, as well as in materials processing science and sensor fabrication to promote the advancement of high-performance room-temperature gas-sensing materials. Moreover, it is expected that the microfabrication industry will adopt 3D printing technologies, which will reduce manufacturing time, waste generation, and cost of the final product. Finally, the IoT is considered a vital environmental engine for developing future sustainable smart cities and will be increasingly used as an environmental monitoring solution.

### 3.2. Humidity Sensors

Humidity sensors are a particularly relevant and unique kind of sensor among gas sensor probes. The term “humidity” is employed to describe the quantity of water vapour in a gas. Humidity impacts all life forms, processes, and materials, and so researchers are always searching for ways to develop stable, reproducible, and durable humidity-sensing materials that display a rapid response and recovery time, high linearity, and ease of connection to control units. The measurement and control of humidity are important in many fields, including industry (e.g., to monitor packaged food quality, control drying processes, or measure the relative humidity in manufacturing operations, including plywood, gum, or paper production plants), agriculture (e.g., dew prevention and monitoring of soil relative humidity), meteorological analysis and forecasting, aviation services, and medical applications (e.g., respiratory equipment, incubators, sterilizers, and pharmaceutical processing).

Polymers and polymer composites possessing hydrophilic properties have found extensive application in humidity sensors, employing various underlying principles. Mechanisms such as hydrogen bonding, intramolecular or electrostatic interactions, chemical bonding, and hydrophilic and hydrophobic interactions contribute to the sensitivity of humidity-sensitive materials. Specifically, polymeric systems reliant on changes in dielectric constant (e.g., capacitive humidity sensors) or conductivity (e.g., resistive humidity sensors) in response to water vapour are among the most prevalent materials in polymer-based humidity sensors.

Regarding capacitive humidity sensors, these modify their dielectric permittivity in response to alterations in humidity and have many advantages, e.g., they can operate in high-temperature environments, are easily and rapidly manufactured, display long-term consistency, and can include a variety of sensing polymers such as polyamides, polyesters (e.g., PET), cellulose acetate butyrate (CAB), etc. On the other hand, among resistive humidity sensors, crosslinked polyelectrolytes containing hydrophilic groups (e.g., -COOH, -SO_3_H, and -N^+^(R)_3_Cl, etc.) and conjugated polymers (copolymers of hydrophilic and hydrophobic polymers) have been widely used. The versatility and control over their structure allows for the tailor-made properties of humidity-sensing devices, making it possible to tune their sensitivity, resistance to high-humidity atmospheres, working range of relative humidity, long-term stability, and response time.

As observed for other gas sensory devices, nanoparticles have piqued the attention of researchers in recent years due to their prominent properties (e.g., stability, wearability, and potential use in electrochemical-sensing implementations). In this context, hydrophilic polymer–metal oxide composites have been lately exploited for humidity-sensing applications including CuO, ZnO, graphene oxide (GO), and carbon nanotubes composites for both capacitive and resistive-type sensors.

As stated above, there is currently a variety of humidity measurement tools based on a wide range of materials functioning on a wide range of principles, environments, and applications. However, some obstacles remain to improving sensor efficiency and response quality in real-world situations. The selection of the appropriate sensing material, and the sensors’ layout and production system both play a key role in their commercial implementation [41].

### 3.3. Ion-Selective Sensors

The accurate and timely detection of different types of ions is crucial for safeguarding both the environment and public health by complying with regulatory standards, implementing effective environmental management strategies, and ensuring drinking water safety. 

Innovative sensors, incorporating novel materials and sophisticated detection mechanisms, are revolutionizing the monitoring and selective response to ions, thereby enabling more informed decisions and actions in medical, environmental, and industrial contexts.

Ion-selective sensors have been designed using polymers as either a conductive component or as the matrix of an electrically conductive system. When these arrangements encounter the targeted analytes, an ionic exchange or interaction takes place, which is read as an electrical signal. The response and selectivity of ion-selective sensors depend on the composition of the polymeric material and is mainly owed to the presence of ionophores that reversibly bind specific targets. Such ionophores can be very different in nature and include organic salts, crown ethers, and other macrocycles.

Alternatively, the development of electrochemical sensors based on PNCs is a highly useful tool for detecting ions, offering an alternative to traditional laborious and costly analytical methods. Additionally, these sensors provide sensitive and real-time monitoring of ions, particularly heavy metals. However, challenges persist in achieving commercial viability, such as ensuring uniform dispersion of nanofillers and simplifying production processes. Therefore, ongoing research is focused on novel synthesis methods and efficient integration of nanoparticles into conductive polymers to enhance sensor performance. Moreover, the development of nanocomposites in biopolymers aims to provide a non-toxic, biodegradable, and biocompatible option. The ultimate goal is to create advanced, cost-effective, and user-friendly sensors for widespread application in various fields [42].

### 3.4. pH Sensors

The pH denotes the concentration of hydrogen ions in a solution, determining its acidity or alkalinity. The surrounding environment significantly influences the course of chemical reactions, thus underscoring the importance of pH measurement and regulation in chemistry, environmental science, and biochemistry. Moreover, the assessment of blood pH holds substantial significance in hospital contexts, serving as a pivotal indicator of a patient’s physiological condition. Various potentiometric pH sensor devices employing polymers have been devised in this realm, including commercially available blood pH sensors [43]. However, these sensors cannot be used in vivo and present several drawbacks, such as reduced sensitivity and lifetime.

In this sense, different pH sensors based on pH-induced reversible changes in optical properties (e.g., absorbance, reflectance, fluorescence, and refractive index) have been developed in past decades because they offer advantages in terms of size, costs, and response time compared to traditional potentiometric techniques. Moreover, newer optical methods offer promise for miniaturization and continuous in vivo measurements, and remote sensing can be achieved with optical pH sensors by transporting the optical beam with optical fibres.

Most optical pH sensors are prepared by immobilizing a pH-sensitive organic dye or fluorophore in a polymer matrix either by adsorption or physical entrapment, which usually leads to leaching and gradual loss of dyes, or by covalent attachment of the dye molecules, which involves a long, complex, and tedious procedure. However, the recent use of CPs in the preparation of such optical pH sensors has eliminated the need for organic dyes, therefore obviating the abovementioned problems. Amongst various polymers, PANI has been described in many research papers as the most suitable for the reversible and optical measurement of pH in the range of 2–12 in aqueous media. Poly(*p*-phenylenediamine) (pPD) has also been revealed as a promising material for its use in modern pH sensors owing to its photoconductive properties.

Alternatively, pH sensors based on poly(vinyl alcohol) (PVA) and poly(acrylic acid) (PAA) hydrogels are also known. The deformation by swelling caused by the increase or decrease in pH generates differences in electrical resistance values, producing a measurable electric impulse.

As observed for most types of sensors, future investigations seek miniaturization, ease of use, and lower production costs. Moreover, as already mentioned, there is a demand for continuous in vivo measurements of blood pH.

### 3.5. Heavy Metal Ions

Remarkably, advances in sensor technology for ion detection have focused considerable attention on heavy metal ions, owing to their heightened toxicity, non-degradability, bioaccumulative nature, and adverse impact on both the environment and human health. The rapid development of modern industrialization has led to the release of heavy metals into the environment at unprecedented levels, posing a serious threat. Therefore, the detection of these species can lead to the early identification of release events, which can lead to early mitigation activities. For example, it is well known that heavy metal ions are oftentimes found in waste effluents from the mining and tannery industries. The release of these contaminants into the environment has had a significant impact on human health and on various aquatic species that could be avoided by the early detection of the released pollutants.

Polymeric sensors offer a solution for detecting and even removing toxic cations from aqueous environments, with mercury being one of the most discussed heavy metal contaminants. Mercury poisoning can lead to serious nervous system dysfunction and illnesses that can ultimately lead to death. The sensitivity, structural stability, and customizable detection properties of polymeric sensors make them well-suited for this purpose. They provide precise sensing and efficient separation by integrating specific binding sites for mercury within their structures and can provide not only an electrochemical output but also allow a visual detection through the evolution of colour or fluorescence [44,45]. Analogous ion-selective sensors have been developed for the detection of other heavy metal ions, including Cd^2+^, Cu^2+^, Pd^2+^, Ag^+^, Al^3+^, Pt^4+^, etc.

Some challenges in developing polymeric sensors for detecting and removing toxic cations include potential interference from other compounds, ensuring selectivity, maintaining durability in harsh conditions, scalability of manufacturing processes, and achieving cost-effectiveness. Overcoming these hurdles is crucial for widely and effectively implementing these sensors in various applications.

### 3.6. Temperature Sensors

Temperature is a fundamental environmental parameter influencing various processes and phenomena in nature and human activities. Therefore, the development of temperature-sensitive sensors is crucial for monitoring and controlling temperature-dependent systems, ensuring optimal performance, safety, and efficiency. Temperature sensors typically consist of temperature-sensitive materials that exhibit detectable changes in their properties, such as electrical resistance (i.e., thermistors), capacitance, solubility, swelling behaviour, or optical characteristics, in response to temperature variations. By accurately measuring these changes, temperature sensors provide valuable information for a wide range of applications, including climate monitoring, industrial processes, geotechnical monitoring, medical diagnostics, appliances, air conditioning systems, and consumer electronics.

In this context, polymer-based temperature sensors offer several advantages, such as ease of fabrication, cost-effectiveness, flexibility of design depending on their intended application, and ability to work in harsh environments where traditional sensors might be incompetent. Moreover, a proper selection of the polymeric material and sensor configuration allows for tailored temperature sensitivity and range. Conventional temperature measurement devices like thermocouples, thermistors, and resistance temperature detectors often encounter inaccuracies stemming from electromagnetic interferences, limiting their effectiveness in applications requiring high precision. CPs and their composites have been widely used for temperature assessment to avoid such limitations. 

Fluorescent thermometers based on polymers have been effective in monitoring temperature since their creation in 2003 when being able to work within microsized domains (e.g., biological cells) or harsh environments (e.g., high voltage), where conventional electrical thermometers are impractical. The key factor lies in the proper selection of the materials, so they achieve aggregation of the polymer chains above their lower critical solubility temperature, which results in the conversion of the microenvironment of the dyes from hydrophilic to hydrophobic triggering an increase in the quantum yield and an easily detectable and measurable fluorescent signal [46].

As an example of their versatility, temperature-sensitive polymers have also lately been incorporated in biomedical applications, particularly in drug delivery and tissue engineering. Regarding the former, these materials can be designed to become more soluble at the higher temperatures found in tumours. By exploiting this property, drugs can be encapsulated in such temperature-sensitive polymers and delivered directly to cancer cells, minimizing damage to healthy tissue. Moreover, polymeric colourimetric sensors are increasingly being integrated into food packaging materials, given their ease of use and ability to inform about the thermal history of food, including temperature fluctuations and thawing and tempering cycles that indirectly affect freshness and food safety. Although these sensors do not directly measure food conditions, they provide useful information about its potential quality degradation.

### 3.7. Mechanochemical Force Sensors

This special kind of sensor, capable of harnessing mechanical forces to trigger chemical reactions, has emerged as a promising avenue as it can be applied in a wide range of fields, from IoT, bioelectronics, and wearable electronics to fluid-based manufacturing processes such as bioprinting.

Mechanochemical sensors utilize mechanically induced bond cleavage or rearrangement within polymers containing specific mechanophores. These mechanophores are strategically integrated to impart intelligent functionalities to the materials. Detection of bond cleavage or rearrangement events occur in diverse settings, such as solution environments, flow fields, exposure to ultrasound, and, notably, in the solid state under compression or elongation forces [47,48].

Overcoming the current challenges in the development of mechanochemical force sensors will represent a leap in their application potential. Obstacles such as achieving reproducibility, improving sensitivity, and shortening response times are still pending research. Moreover, incorporating these materials into wider technological applications requires establishing standardized calibration and testing procedures, particularly for emerging electrical signal outputs. The viability of these materials in applications requires further understanding of the interactions within the host polymer matrix and the development of systems that allow activation without damaging it. Advances like integrating fillers, establishing double networks, or utilizing micelle-based structures within hydrogels are proposed to address these challenges. 

**Figure 7:**
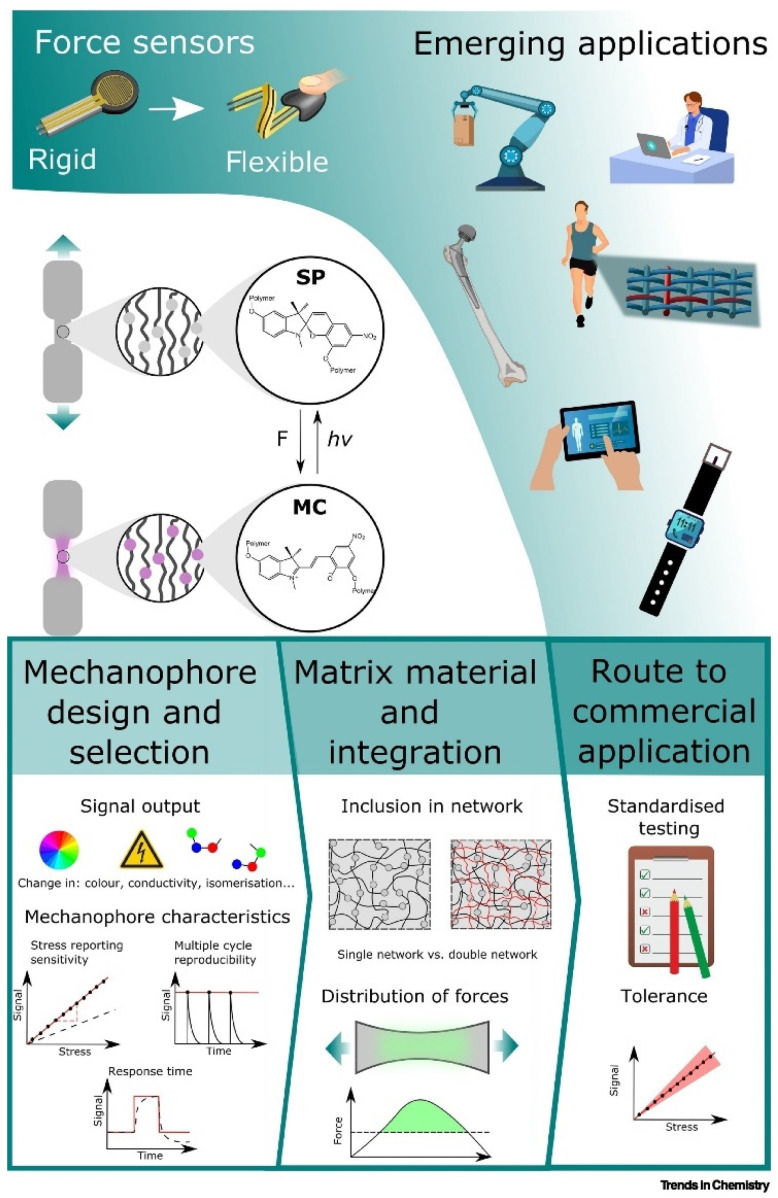
Summary of the development phases of flexible force sensors using mechanosensitive polymers (reprinted from [49] under CC BY 4.0 license).

On the other hand, the design of mechanophores must continue to progress and advance. Tackling questions such as developing mechanophores with reversible electronic properties and ensuring resistance to undesired stimuli is vital for the progression of the field. Figure 7 shows the design of mechanophores and the process of development and implementation into the market [49].

It is worth noting that these innovative materials have the potential to revolutionize a wide range of industries, from healthcare to manufacturing, by offering practical and efficient solutions for the detection and precise measurement of mechanical forces. For example, pressure sensors are advancing rapidly, presenting potential uses in health monitoring, tactile systems [50,51], electronic skin, and biosensing. However, their transition from the laboratory to commercialization is marked by a series of technical and logistical challenges that require a multidisciplinary approach and careful consideration. In this context, cost-effective scale-up, manufacturing processes, and the establishment of required standards and adequate technological infrastructure are obstacles and opportunities that mechanochemical polymer research faces in its path toward commercial implementation. 

Future investigations seek to develop novel polymer materials endowed with adaptable electrical and mechanical properties, heightened sensitivity to diverse pressure ranges, and seamless integration with other sensors to unlock multifaceted functionalities [52].

### 3.8. Organic Compounds Sensors

Organic aromatics are another kind of environmental pollutant with potential danger due to their poor biodegradability, allowing them to persist for long periods, thus leading to their accumulation. For many years, they were released into the environment ignoring the consequences this would have on wildlife, environmental, and human health. In the case of detecting organic compounds, it is important to implement effective environmental management strategies and comply with health regulatory standards to protect the environment and public health. Advances in sensor technology are evolving to enhance the capability for real-time monitoring and decision-making in response to these compounds, aiming to mitigate pollution and food safety more efficiently. Newly developed polymer-based assays for the detection of environmental pollutants rely on electrochemistry, fluorescence, colourimetry, and UV-vis spectroscopy.

**Figure 8:**
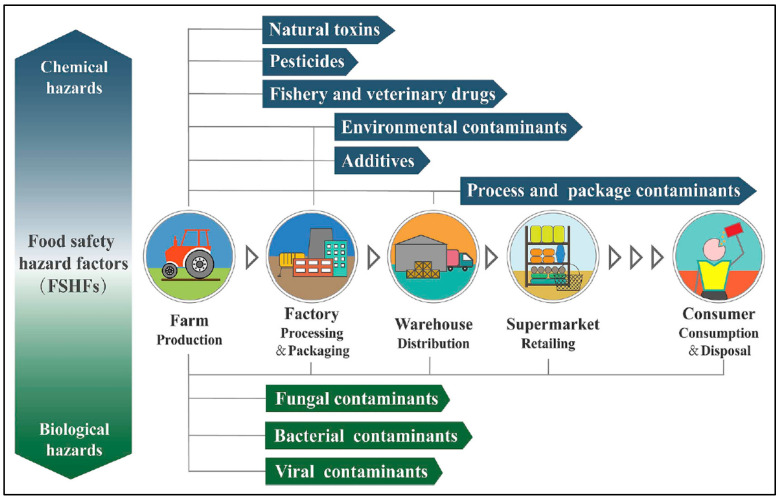
A selection of Food Safety Hazard Factors (FSHF), (reprinted from [12], Copyright (2024), with permission from Elsevier).

Due to their high specificity and stability in adverse conditions, sensory polymers are replacing traditional detection methods, offering sensitive and real-time monitoring of contaminants such as pesticides, insecticides, plastics, and Food Safety Hazard Factors (FSHF), such as certain chemicals (Figure 8). In this context, compounding refers to the unintended accumulation or combination of substances, often resulting in adverse effects. A significant historical example is the widespread use of dichlorodiphenyltrichloroethane (DDT) as an insecticide during the mid-1940s. Another notable instance involves the inclusion of bisphenol A (BPA) in the production of polycarbonate plastics, commonly found in beverage bottles and various containers, which could potentially lead to human ingestion. 

The polymeric sensors most widely used in the detection of organic compounds are MIPs, and they have been extensively studied to enhance food safety due to their ability to efficiently, sensitively, and cost-effectively detect molecules ranging from small molecules to toxins. For example, optical sensors based on MIPs offer a solution for selective, sensitive, and cost-effective detection of pesticides in food systems, making a significant advancement in monitoring pesticide residues posing health risks, using fluorescence, colourimetry, and spectroscopy. Additionally, MIPs/NCPs have been utilized to create electrochemical, optical, and mass-sensitive sensors, representing a significant advancement in food analysis technology aimed at achieving specificity and sensitivity [12,21,53].

However, challenges persist in the field of sensory technology as it integrates into advanced detection platforms like electrochemical and optical sensors. Improving sensor performance by incorporating conductive nanoparticles and developing bio-based, biodegradable polymer matrices for organic compound detection is a significant focus. Future research aims to enhance sensor cost-effectiveness and market integration through novel synthesis methods, while also expanding the detection range and offering portable solutions for rapid on-site analysis. Additionally, efforts are underway to develop user-friendly sensors that eliminate the need for specialized personnel, thereby replacing current laborious analytical techniques.

#### 3.8.1. VOCs 

Volatile organic compounds (VOCs) are organic chemicals known for their high vapour pressure at room temperature. They encompass a diverse range of substances, classified based on functional groups like aliphatic hydrocarbons, oxygenated hydrocarbons, halogenated hydrocarbons, carbonyl compounds, and aromatic hydrocarbons, among others. Some of them are even capable of detecting harmful volatile organic compounds such as isocyanates or chlorinated gases, causing respiratory irritation, neurological effects, and long-term cancer risks [41].

The detection of VOCs has numerous applications, including environmental monitoring to assess outdoor air quality, industrial safety to detect gas leaks in chemical plants or factories, and indoor air quality monitoring in buildings, homes, and workplaces. Various technologies and devices are used to detect and measure VOCs. These include photoionization detectors (PID), thermal conductivity detectors, gas chromatography (GC) with flame ionization detectors (FID) or mass spectrometry (MS), electrochemical sensors, and other sensor-based technologies. For their part, polymeric sensors play a critical role in gas detection due to their high sensitivity, enabling early and accurate identification of substances across diverse environments. Their customizable nature ensures enhanced selectivity for specific applications, while their lightweight, flexible design facilitates cost-effective deployment in extensive sensor networks for real-time monitoring. Monitoring applications often involve complex mixtures of vapours and gases found in environments, food products, and exhaled breath, necessitating sensitive materials that can ensure selectivity and minimize cross-sensitivity.

Moreover, due to their negative impacts on human health and the environment, there are regulations and standards that limit VOC emissions in different industrial sectors and establish indoor and outdoor air quality standards. Therefore, ongoing research and development in the field of VOC detection is imperative to improve the sensitivity, selectivity, response time, and portability of detection devices. Exploring the design of multiple recognition combinations, including MIPs and the application of dummy templates, may be beneficial. MIPs can collaborate with other advanced technologies, such as nanotechnology, artificial intelligence, and lab-on-a-chip technology. This includes the development of miniaturized sensors, more affordable sensor technologies, and real-time detection systems [12].

#### 3.8.2. Drugs

A noteworthy aspect to emphasize is the detection of drugs within organic compound polymeric sensors, owing to the potential health risks they pose to consumers. Consumption of food containing drug residues can lead to adverse effects such as allergic reactions, antibiotic resistance, and other health complications. Moreover, it raises concerns about the integrity of the food supply chain and compliance with food safety regulations, given the risky contamination by antibiotics resulting from agricultural and veterinary practices. 

Luminescent sensors have emerged as prominent tools in drug detection due to their ability to provide rapid, specific, and sensitive detection, making them a crucial asset in combating antibiotic contamination in the food supply chain. Meanwhile, MIPs have demonstrated potential by showing selectivity and sensitivity. However, further research is needed to achieve quick response times and cost-effectiveness and to operate without the sample pre-treatment procedures [54].

It is worth noting that detecting drug molecules poses a significant challenge due to their low concentration in biofluids, such as sweat. Drug molecules are typically present in extremely low concentrations in sweat compared to other biological fluids like blood or urine. This makes precise and reliable detection more difficult, as sensors must be highly sensitive and selective to detect the minute concentrations of drugs in sweat. Additionally, the sweat matrix’s complexity, containing various organic compounds, can also hinder specific drug molecule detection. Therefore, improving technologies and wearable devices, such as sweat-sensing textiles, for drug molecule detection represents a significant challenge in current research [55].

### 3.9. Biomolecules

Polymeric sensors signify a notable leap forward in diagnostic technology, providing high sensitivity, selectivity, and rapid response times in detecting a wide range of analytes. These sensors leverage the characteristics of polymers, including their structural adaptability and ability for functionalization, to establish precise interactions with target biomolecules. The integration of specific sensory units within the polymer matrix enables direct conversion of chemical interactions into measurable signals, thereby streamlining the quantitative analysis of samples.

Recent advances focus on the detection of biomarkers in saliva, which constitutes a non-invasive approach to health monitoring. These innovative sensors provide a comprehensive source of information on both physiological and pathological conditions, circumventing the discomfort and invasiveness typically associated with traditional blood tests. The utilization of polymer sensors for saliva analysis offers valuable insights into the body’s health condition, as saliva, as a diagnostic fluid, contains a diverse array of analytes, encompassing hormones, enzymes, antibodies, and nucleic acids. This rich composition renders it an ideal medium for early disease detection, monitoring health status, and facilitating personalized medicine initiatives [54].

As we can see, this technology benefits the healthcare sector but also faces several challenges. One major obstacle is the potential for interferences in sensor performance caused by the diverse substances comprising the saliva composition. Moreover, the low concentration of specific biomarkers in saliva requires the creation of exceptionally sensitive sensors for detecting analytes at nanomolar or even picomolar levels. Additionally, ensuring the stability of polymer sensors in the oral environment, where factors like pH and temperature can fluctuate significantly, is essential for obtaining reliable measurements [8].

The future of polymer sensors applied in saliva exhibits significant promise and presents several avenues for ongoing research. Firstly, to notably augment the utility of saliva as a diagnostic medium, research endeavours should focus on the development of multiplexed sensors capable of simultaneously detecting multiple analytes. Furthermore, the advancement of smart polymers capable of responding proportionally to fluctuations in analyte concentration offers prospects for dynamic health condition monitoring. Moreover, the exploration of biodegradable and bio-based polymers represents a pivotal step towards addressing environmental concerns related to sensor disposal, thereby enhancing the applicability of these sensors in the realm of personalized medicine.

#### 3.9.1. Proteins

Protein detection and quantification are critical in the biomedical field, offering valuable insights into various biological processes. This includes assessing protein presence, levels, structure, activity, and interactions. Materials used for protein detection must meet specific criteria such as ease of mass production, biological durability, chemical versatility, cost efficiency, and portability. Polymers are highly suitable for these applications and are preferred materials in various biological detection platforms. Polymers serve as active or passive components in protein detection methods, offering diverse physical properties, including optical, electrochemical, electrical, mass-sensitive, and magnetic characteristics. Research focuses on leveraging polymer properties to enhance sensitivity, selectivity, and performance in protein detection systems [56,57].

**Figure 9:**
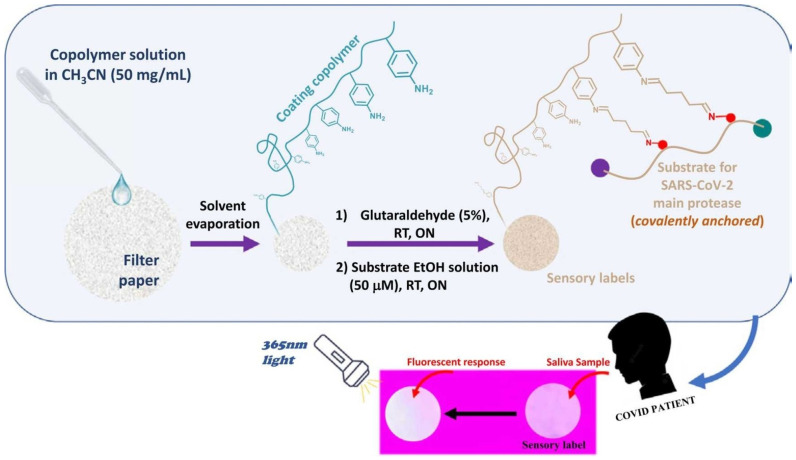
Polymeric sensory film for the detection of SARS-CoV-2 in saliva based on a peptide substrate containing a FRET pair (fluorophore and quencher) to detect the Mpro protein (reprinted from [58] under CC BY 4.0 license).

An exemplary application of these sensors is the detection of SARS-CoV-2 based on a peptide substrate. A polymer-coated cellulose material was utilised instead of using traditional invasive testing methods, which require complex sample collection. This material contained a fluorogenic peptide substrate of the virus’s main protease (Mpro), which was covalently immobilized. Upon contact with saliva from COVID-19-positive patients, these sensory labels displayed fluorescence. This innovative approach, illustrated in Figure 9, highlights the potential of polymer chemosensors in early disease detection, providing a straightforward, non-invasive, and rapid testing solution [58]. 

#### 3.9.2. Nucleic Acids and Other Metabolites

Detection of specific DNA sequences is crucial for predicting certain human genetic diseases and, therefore, for early clinical disease diagnosis. In recent years, electrochemical DNA sensors have gained attention due to their simplicity, portability, high sensitivity, and selectivity, alongside their rapid response and low manufacturing cost, making them ideal for point-of-care (POC) applications and patient monitoring. Although significant progress has been made, challenges remain in the sensitivity and reproducibility of these sensors, and considerable research is currently being conducted on the use of smart sensors in this area. In addition to the challenges mentioned above, the development of robust biosensors for DNA detection faces several hurdles. One such challenge is the need for enhanced sensitivity and specificity to ensure accurate detection of target DNA sequences, particularly in complex biological samples with low target concentrations. Moreover, the integration of electrochemical DNA biosensors into practical diagnostic devices requires addressing issues related to sample preparation, stability, and shelf-life. Ensuring the reliability and reproducibility of sensor performance over time and under varying environmental conditions is essential for their successful deployment in clinical settings. Furthermore, the translation of research findings from bench to bedside necessitates rigorous validation studies to evaluate the clinical utility and accuracy of these biosensors. Collaborative efforts between researchers, clinicians, and regulatory agencies are crucial for streamlining the regulatory approval process and ensuring the safety and efficacy of these diagnostic technologies.

Despite these challenges, electrochemical DNA biosensors’ continued innovation and development hold immense promise for revolutionizing clinical diagnostics and personalized medicine. By overcoming existing limitations and addressing emerging needs in healthcare, these biosensors have the potential to impact disease management and patient outcomes significantly.

On the other hand, we encounter polymer sensors capable of detecting metabolites. The detection of intermediates and byproducts of metabolism in blood, such as glucose, cholesterol, hydrogen peroxide, urea, lactate, and creatinine, is essential in human disease diagnosis and health monitoring, as they are indicators of various diseases. For instance, blood glucose concentration is an important indicator for diabetes control, while high cholesterol levels may be a risk factor for cardiovascular diseases. Hydrogen peroxide can serve as a marker for oxidative stress, and creatinine is useful for assessing kidney function. For example, the concentration of lactate is associated with various biological processes, including tumour cell metastasis and head trauma. Therefore, the detection and quantification of metabolites are of great importance for understanding and diagnosing a wide range of medical conditions. 

Despite advances in sensor technology, challenges persist in implementing these devices in clinical settings. Enzyme-based electrochemical sensors often experience signal drift due to variability in patients’ physiological conditions and interference from other substances present in the biological sample. These interferences can lead to inaccurate measurements and affect the reliability of the results. Similarly, while optical sensors offer advantages such as continuous monitoring capability and label-free fabrication, challenges remain in terms of sensitivity and specificity. Continuing research efforts focus on optimizing these devices to achieve precise and reliable detection under diverse clinical conditions.

In summary, metabolite detection using polymeric sensors holds great promise for diagnostic medicine and health monitoring, but still faces challenges in terms of accuracy, stability, and specificity in clinical applications [8].

### 3.10. Pathogens Sensors

Detection of bacterial pathogens such as *Salmonella*, *Vibrio*, *Shigella*, *Escherichia* (e.g., *E. coli*), *Staphylococcus*, and *Yersinia* is extremely important to identify early stages of infection and prevent transmission in a population to control outbreaks, as well as to initiate treatment as soon as possible. This has become especially crucial with the increasing number of infections caused by multidrug-resistant bacteria. With technological progress, various approaches have been developed over the last decade to overcome the limitations of traditional phenotypic identification methods in terms of increased sensitivity, selectivity, and reduced analysis time. Some of these methods include real-time polymerase chain reaction (real-time PCR), matrix-assisted laser desorption ionization-time of flight mass spectrometry (MALDI-TOF MS), and immunological assays such as ELISA. However, these methods are costly, time-consuming, and require the use of laboratory facilities and specialized technical personnel, which limits their application. Therefore, the development of low-cost and user-friendly detection platforms for pathogenic bacteria is generating significant interest.

Polymeric materials are attractive candidates for the development of detection platforms as they can provide early insights into contamination or infection. Conducting/conjugated polymers have also garnered significant attention in the development of chemical and biological detection strategies due to their high electrical conductivity, low cost, and good processability [8].

These sensors, with their high specificity and sensitivity, are emerging as crucial tools in the timely diagnosis and management of cancer. Furthermore, their application extends beyond medical fields to address pressing concerns in food quality and safety. By detecting pathogens, these polymeric materials enable proactive measures to be taken, effectively preventing foodborne illnesses, and safeguarding public health.

Hence, cancer bio- and chemosensors, especially those utilizing electrochemical methods, are gaining recognition for their outstanding sensitivity and selectivity in detecting cancer biomarkers. While MIP-based sensors show promise due to their cost-effectiveness, existing challenges in optimizing performance require further investigation.

Potential advancements encompass the development of innovative MIPs integrated with microfluidics for portable diagnostics, smartphone connectivity for rapid self-testing, and aptamer hybridization for multiplexed biomarker detection. These developments collectively enhance the potential of these sensors to transform cancer diagnosis and therapy [58].

A similar principle holds true for rare diseases like cystic fibrosis, where the use of polymeric sensors marks a significant advancement in personalized medicine. These cutting-edge sensors play a pivotal role in improving patient outcomes by facilitating early detection and customized treatment approaches, offering a rapid, non-invasive, and economical method for monitoring and managing patients’ quality of life [59].

It is worth mentioning that the development of bacterial detection platforms has also been investigated. In the case of bacteria, distinguishing and detecting live or dead bacteria is also a major challenge. There are several challenges associated with different types of optical sensors that require sample labelling with various fluorescent tags, increasing detection time and cost and potentially damaging cell physiology. When designing biosensors to detect bacteria, the requirements include excellent specificity, label-free operation, viability distinguishability, cost-effectiveness, compactness, portability, and ease of operation, as well as fewer sample preparation steps. On the other hand, aptamer-based biosensors offer advantages such as stability against temperature and pH changes. In recent years, bacteriophages have been utilized as bio-recognition elements due to their specificity, stability, sensitivity, selectivity, rapid response, easy availability, and low cost.

Currently available biosensor designs have detection limits ranging from fM to aM. Therefore, there is a need for a highly sensitive biosensing platform that can detect at very low concentrations in the range of pM, zM, and yM. On the other hand, power consumption is a significant challenge when making implantable biosensors. In this context, research is focused on the utilization of a self-powered device. Among various optical biosensing platforms, only colourimetric and fluorescence-based sensors are commercialized. Sensors based on SERS, interferometric, and plasmonic techniques are still in the development stage. It is important to mention that commercialized applications of plasmonic sensors are limited to small molecules, cancer cells, and chemical detections due to the shallow penetration depth of the electromagnetic field into bacteria. One of the significant issues is that the quantification associated with the number of bacteria detected by the sensor relies on calibration, which can be improved with labels [17].

Early and cost-effective pathogen detection through polymeric sensors poses significant challenges in environmental care and food safety. These challenges include ensuring the sensors’ sensitivity and accuracy in detecting a wide range of pathogens, optimizing their performance in diverse environmental conditions, and developing cost-effective solutions that can be easily integrated into existing food safety protocols. Additionally, there is a need to address potential limitations in sample preparation and handling to streamline the detection process and improve overall efficiency. Overcoming these challenges is essential to harnessing the full potential of polymeric sensors to enhance environmental monitoring and ensure food safety.

### 3.11. Multifunctional/Multitasking Sensors

The development of multitasking materials represents more than just an incremental progress; it marks a revolutionary boom that has the potential to expand the applicability of conventional sensors greatly. Thanks to their high sensitivity and specificity, these cutting-edge polymers are expected to react and respond to a diverse range of changes or stimuli in real time. A major challenge in the sensory polymer technology field has been crafting sensors capable of selectively responding to multiple stimuli simultaneously. 

**Figure 10:**
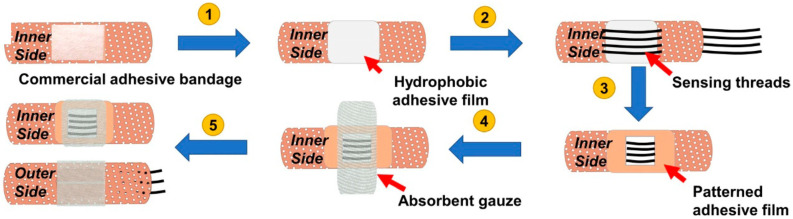
A schematic illustration of the sensor for multiplexed perspiration analysis. Threads are connected to reusable wireless readout electronics for real-time data acquisition and collection from potentiometric (for pH, ammonium, and sodium sensing) and amperometric (for lactate) sensing (reprinted from [60] under CC BY 4.0 license).

These sensors have the potential to incorporate numerous sensing capabilities into a unified platform, allowing for the detection of a wide range of analytes, from physical parameters such as temperature and pressure to chemical and biological molecules (Figure 10). This integration promises to streamline operations across diverse industries by providing more exhaustive monitoring capable of implementing smarter and more responsive technological solutions and delivering comprehensive environmental assessments, thereby strengthening safety and prevention measures [3]. 

Sweat sensors are one of the most representative examples of multitasking sensors. These sensors facilitate continuous, real-time, non-invasive detection of sweat analytes, affording insights into human physiology at the molecular level. They have garnered substantial attention for their promising applications in fitness tracking and health monitoring in high-performance sports for athletes, and in disease diagnosis and medical monitoring. Recent developments in soft microfluidics, multiplexed biosensing, energy harvesting devices, and materials have advanced the compatibility of sweat-sensing platforms. Sweat is rich in biomarkers: electrolytes (e.g., sodium, potassium, ammonium, calcium, and chloride), metabolites (e.g., alcohol, glucose, and lactate), trace elements (e.g., iron, zinc, and copper), small molecules (e.g., cortisol, urea, and tyrosine), neuropeptides, and cytokines. Compared with blood or other body fluids, the biomarker content in sweat is lower, presenting a challenge that necessitates significant advancements in sweat collection and transport to achieve reliable and precise real-time monitoring. In terms of sweat stimulation and collection, the integration of an iontophoresis device makes it possible to obtain sweat via chemical stimulation while the body is at rest, and the incorporation of microfluidics makes the collection and analysis of sweat faster and more efficient. This type of sensors for the simultaneous detection of multi-analytes in sweat are rapidly evolving, achieving a shift towards simultaneous non-invasive monitoring of a broad range of biomarkers [8,55,61].

Therefore, further research is still needed to deepen the accuracy of multi-responsive sensors and improve the data collection that yields varied responses to a single stimulus. The current challenges faced by these materials can be approached from various perspectives, ranging from engineering and materials science to chemometrics and data analysis, as well as AI and advanced algorithms, to enhance pattern recognition and distinguish between similar stimuli, enabling the differentiation and quantification of multiple analytes [28].

Synthesis and design of multitasking sensors are progressing towards materials with multiple sensory capabilities. For instance, sensory arrays are developed by assembling sensors for each tailored specific stimulus or hybrid materials that integrate different reactive components within a polymeric matrix, such as metallic nanoparticles providing magnetic sensitivity and organic compounds for chemical reactivity. Moreover, the properties of these sensors formed by MIPs with selective binding sites within the polymers are also being enhanced. When combined with electrical or optical signals, they enable the detection and quantification of multiple specific molecules. Additionally, smart nanocomposites are being developed to detect and respond to various stimuli such as light, pressure, and chemical interactions. These advances will enable cross-response networks by connecting sensors with overlapping detection capabilities to identify complex stimuli through interconnected reactions [62].

## 4. Smart Devices

Finally, the boom of smart polymeric structures and sensor arrays has represented a significant advancement towards complex and integrated systems, whereas lab-on-a-chip devices have exemplified the miniaturization and refinement of these technologies. Nanotechnology has played a pivotal role in boosting sensor performance by embedding nanoparticles within polymer matrices, thereby enhancing signal transduction and improving the detection limits. Additionally, the integration of polymer sensors with microfluidic devices and wearable technology has paved the way for new possibilities for real-time analysis of various biomolecules in diverse environments. To address this approach, we will include three overarching sections that cover these topics: response time and transduction processes, and the exploitation of wearables and smartphones for portable and convenient detection and recognition tasks.

### 4.1. Response Time and Transduction

Reducing detection or response time stands out as one of the most significant challenges in sensory technology research, especially regarding identifying dangerous substances like heavy metal ions or explosives [63,64]. The rapid detection of these substances is essential for ensuring safety and minimizing risks, emphasizing the critical necessity for rapid sensor responses in sensor development.

The transduction process, responsible for converting the chemical interaction between the sensor’s receptor and the target analyte into a measurable signal—electrical, mechanical, or optical—is a pivotal aspect for enhancing the speed and efficacy of sensors. By improving this conversion mechanism, sensors can attain more precision, reliability, and sensitivity in detection capabilities. The challenges in enhancing the transduction process are focusing on reducing the response time to achieve quantifiable results and increasing the quality of detection, considering that modifying the microstructure can improve critical parameters. For example, the implementation of foaming techniques may decrease response times and enhance detection and quantification limits due to the increased contact surface area with the analyte [65].

Recent advancements in reducing detection time in a wide range of sensing applications involving polymeric sensors include microfluidic fabrication techniques and Quartz Crystal Microbalances (QCMs). These innovations capitalize on precise fluid-flow control and sensitive measurement of mass changes, respectively, intending to accelerate detection processes. As research in this field continues to progress, integrating such cutting-edge technologies into sensory polymer applications holds the potential to establish new opportunities for response times and transduction efficiency in detecting dangerous substances [66].

### 4.2. Wearables

The polymeric smart devices that have experienced significant development in recent years have been wearable sensors. This surge is primarily attributed to their versatility and applicability across a diverse range of fields. Wearables have emerged as pivotal tools at the forefront of technological innovation, from revolutionizing healthcare and medical diagnosis to enhancing robotics, prosthetics, and virtual reality experiences to even making waves in professional sports and entertainment industries [67]. 

In particular, the development of advanced wearable strain sensors has garnered considerable attention in the field of biomonitoring due to their ease of use, enhanced diagnostic capabilities, and potential for long-term monitoring. Ideal strain sensors should possess key characteristics such as high elasticity, sensitivity, and durability for prolonged use without performance degradation. Combining these requirements into a single sensor is itself a significant challenge. This is why traditional metallic foil or semiconductor strain gauges are not suitable candidates for wearables due to their deformation limitations. In recent years, driven by the increasing demand for these devices, significant advancements have been made by combining materials science, nanotechnology, and microelectronics, meticulously studying material selection and design to balance sensitivity and stretchability. 

Many of these efforts have focused on the well-known nanocomposite polymers (NCPs), aiming to develop flexible energy sources for wearable devices and refine manufacturing processes to enhance reliability and durability in real-world applications [52]. For instance, a typical nanocomposite polymer strain sensor consists of a conductive network for generating responses and an elastomeric polymer to provide flexibility, elasticity, and protective insulation. However, challenges persist regarding material selection, as each conductive nanomaterial presents unique drawbacks, including limited conductivity, electron conduction mechanisms (e.g., disconnection, crack propagation, and tunnelling), and processing difficulties such as aggregation in carbon black and poor adhesion and high cost of metal nanowires. Scalability and cost-effectiveness are prerequisites for commercialization. Hence, recent innovations have focused on fully printed electronics for mass production. Integrating wearable sensors into a complete system with power, signal processing, and communication capabilities is an unmet goal. Further research and collaboration between scientists and industry is needed to overcome obstacles such as reliability, robustness, and power efficiency and to explore attractive features like self-healing, self-powering, and biodegradability [68,69,70,71,72].

**Figure 11:**
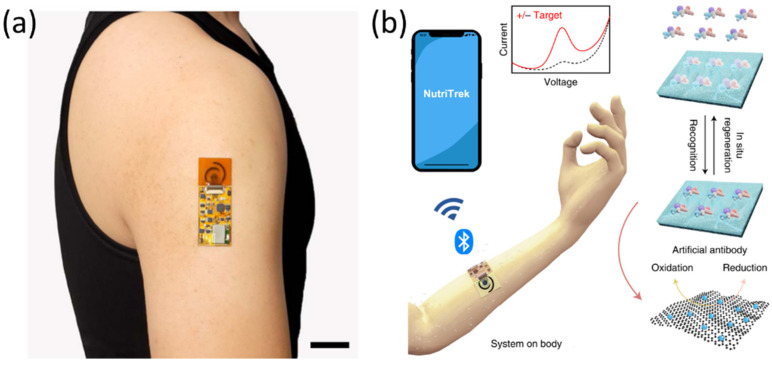
Wearable sweat-sampling electrochemical MIP sensor used for on-site and timely health monitoring: (**a**) wearable sensing patch in practical use and (**b**) components contributing to the sensor and its working mechanism (reprinted from [14] under CC BY 4.0 license).

On the other hand, the utilization of hydrogels and conducting polymer hydrogels (CPH) also represents notable progress in wearable and implantable biomedical sensors, capitalizing on their unique properties related to flexibility, resilience, high mechanical strength, and stretchability, and advanced fabrication techniques. As a result, they are widely used in applications such as e-skin sensors (Figure 11). These innovative sensors have become a growing trend in human health monitoring due to natural skin’s tactile and responsive mimicry [73]. 

The development of sensors that not only detect changes in strain but also conform to the dynamic contours of the human body is crucial for providing accurate and uninterrupted monitoring capabilities. CPHs are particularly well-suited for e-skin sensors because they can be easily customized to specific sensor requirements through simple chemical structure or composition modifications in addition to their inherent physical properties, such as strength and elasticity. This adjustability is a significant advantage, enabling the creation of sensors capable of maintaining high performance under various mechanical strains. However, challenges remain in developing long-lasting, adaptable wearables with self-repair capabilities, heralding a new era in continuous, integrated human health monitoring [1,74].

A particular type of wearable technology deserving special attention is smart sensory textiles. Fibrous textile threads, used as sensors, offer great promise. They can be seamlessly integrated into garments and placed in close contact with the skin. A thread-based sensing platform, in the form of a multiplexed patch, enables continuous on-skin monitoring, providing real-time measurement of key sweat biomarkers like electrolytes, metabolites, and pH. These sensors can be integrated into a patch connected to a miniaturized circuit module for smartphone readout [60].

In this context, glove-based sensors represent a significant advancement by offering a unique combination of functionality and advanced sensing capabilities for various applications. For example, they find utility in crime scene investigation, airport security, and disease control, allowing the analysis of both dry and liquid samples by simply swiping the sensor-equipped glove over the desired surface. These sensors function as adaptable analytical instruments, facilitating the identification of various substances, from drugs and dangerous chemicals to pathogens, across different surfaces. It is worth noting that the integration of glove-based sensors into smart textiles and garments enhances their potential and enables continuous health monitoring without sacrificing comfort, as they seamlessly blend with daily attire. Glove-based sensors offer a non-intrusive alternative, whereas other wearable sensors might pose risks of infection or discomfort with prolonged use. However, the efficacy of these sensors depends on carefully selecting glove sensory materials and conducting nanomaterials, ensuring the requirements of transducer modification techniques. Thus, innovative strategies are being employed to tackle challenges related to material selection, sensor design, and the integration of sensors with textiles, highlighting the versatility and promise of this technology.

It is worth noting another type of sensor in this section, eyeglasses-based sensors. These innovative sensors offer a fascinating perspective on the use of polymers in biomedical applications with the ability of detecting a variety of biomarkers and health conditions in a non-invasive and user-friendly manner [75]. For example, sensors integrated into eyeglass frames could monitor glucose in tears for continuous diabetes management, or even detect biomarkers related to conditions such as high blood pressure or eye fatigue [76]. These kinds of sensors not only improve accessibility and convenience for patients but also open up new possibilities for early detection and personalized health monitoring, thus highlighting the crucial role of polymers in advancing biomedical technology [77].

Additionally, the sustainability of these sensors is a paramount concern, prompting research into methods for responsibly disposing of used sensors in line with Sustainable Development Goals (SDGs). In this context, wearable glove-based technology and smart sensory textiles aim to enhance public safety and health monitoring and address environmental challenges. This advancement will turn wearable sensors into an indispensable part of our daily lives, serving as powerful health and safety monitoring tools. Therefore, continued research and advancements are imperative to further progress in this field [78,79].

### 4.3. Smartphones

Another significant approach in smart devices is the technology that combines sensory polymers with smartphones, emerging as a novel portable detection and recognition method. This type of sensor harnesses the built-in sensors and computational power of smartphones, offering a portable and immediate solution for sensing needs in various fields, like bioanalysis, clinical diagnostics, point-of-care testing, and environmental monitoring. This innovative technology could be considered an alternative to detection devices due to its numerous advantages, including an economical, quickly fabricated, and user-friendly detection system [80].

**Figure 12:**
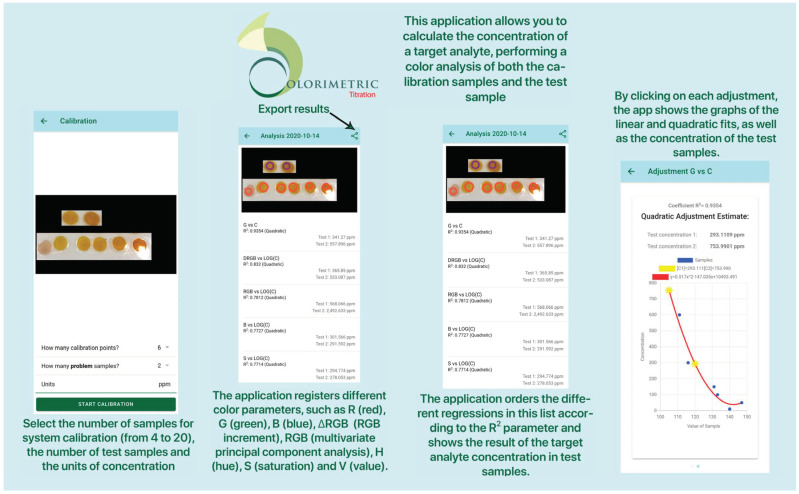
Colourimetric titration: sample of a smartphone app for determining the concentration of a target species based on colourimetric or fluorogenic sensory film [80,83]. It can be freely downloaded from App Store and Google Play.

Characteristics such as simplicity, speed, and broad applicability are greatly favoured by the colourimetric method, as it is capable of transforming these smart devices into versatile analytical instruments for health monitoring and safety assessments [81,82]. The performance of colourimetric sensory polymers in on-site visual detection can be greatly enhanced through smartphone-assisted quantification. For instance, this capability has been demonstrated in the easy nitrite analysis of processed meat using colourimetric sensory polymers and a smartphone app. The method involves obtaining colour parameters from a photograph taken of the test polymeric sensory materials using the smartphone app “Colorimetric Titration”, enabling self-calibration of the system under identical lighting and distance conditions independently from smartphone models. This demonstrates the adaptability and precision of the process without specific lighting requirements. The app can analyse digital colour parameters and make the corresponding adjustments to determine concentration levels based on the best R2 coefficient (Figure 12) [80].

Despite these advantages, certain challenges, such as implementation and market rollout, must be overcome. To achieve this, it is necessary to optimize the sensory performance of sensory polymers and enhance smartphones’ current imaging and computing capabilities.

Furthermore, achieving appropriate detection parameters, high sensitivity, and selectivity of the probe material is essential. To attain satisfactory sensing parameters, adjustments to the chemical structure and nanoscale morphology of the material are necessary. Proper adaptation of the polymer material to the analyte is crucial for achieving optimal sensing capabilities. The key challenge lies in designing recognition elements that exhibit strong and selective affinity towards the analyte. This can be accomplished by controlling the shape of the macromolecule through crosslinking or precise monomer sequence control. Therefore, gels and foldamers are attracting significant interest as sensor probe materials. As technology progresses, these initial hurdles are expected to be surmounted to develop future commercial applications [84].

## 5. Challenges

Sensory polymers have emerged as a revolutionary force in the field of sensing technology, undergoing a significant transformation from simple passive materials to complex systems capable of actively detecting and responding to a wide range of physical, chemical, and biological stimuli. Their historical evolution is characterized by increasing sophistication, expanding from basic chemical detection to the multitasking and highly selective sensors developed today. These advancements have solidified their position in sensor technologies, surpassing the limitations of traditional materials and unlocking functionalities that once seemed unattainable. 

Given the development of polymer-based sensors, which involves various synthesis methods, it is important to consider the strengths and weaknesses of these methods in a concise manner. Each method’s unique advantages and challenges highlight the importance of selecting the appropriate synthesis technique based on specific application requirements [13,25,85]. For instance, free radical polymerization is a widely used method known for its simplicity and the extensive variety of monomers available, which allows the creation of polymers with diverse properties. However, it often results in polymers with broad molecular weight distributions and less control over the final structure, potentially affecting sensor reproducibility and performance. In contrast, MIPs and electrochemical polymerization have emerged to overcome these limitations. MIPs create highly selective polymers with specific cavities formed during polymerization in the presence of a template molecule. This technique provides exceptional selectivity for target analytes but can suffer from incomplete template removal, affecting binding site accessibility and sensor sensitivity. In the case of electrochemical polymerization, it offers precise control over polymer thickness and morphology, making it ideal for creating thin films on electrode surfaces and developing highly responsive electrochemical sensors. Nevertheless, the complexity of the process and the need for specialized equipment can limit its accessibility.

Another method, Sol–Gel processing, enables the incorporation of functional groups into the polymer matrix, enhancing sensitivity and selectivity. This method is beneficial for creating tailored materials; however, it can be time-consuming and may involve harsh conditions, limiting the range of compatible monomers.

Additionally, block copolymer self-assembly and layer-by-layer (LbL) assembly utilize self-assembly properties to create nanostructured materials with specific sensing capabilities or tuneable and highly responsive sensor surfaces for precise control over film thickness and composition. While these methods allow for designing responsive materials for various stimuli, the labour-intensive nature of the process can be time-consuming and may not be suitable for large-scale production. In the same vein, electrospinning produces ultrafine polymer fibres with a high surface-area-to-volume ratio, enhancing sensor sensitivity. However, achieving consistent fibre production requires precise control over spinning parameters. In the case of photopolymerization, it is particularly useful for creating patterned surfaces and microstructures. Nevertheless, the need for light-sensitive monomers and limitations in light penetration depth can restrict its application to certain types of sensors. The versatility of sensory polymers, combined with their ease of fabrication and tuneability, continues to drive innovation in sensor technology.

Such innovations originate from the intersection of various disciplines, including chemistry, physics, materials science, and engineering. This interdisciplinary approach fuels ongoing research efforts that consistently expand the possibilities of what sensory polymers can accomplish across various sectors. These innovative materials have generated a new era of smart materials, showcasing their potential for selective, reversible, and tailored interactions with external stimuli. Their versatility has led to advancements in the biomedical sector, where sensory polymers now play a critical role in diagnosis and health monitoring, offering non-invasive options for patient care, and pioneering wearable technology that synergizes with personal devices for health tracking. The environmental sector has also witnessed significant progress in monitoring and pollution control, even in extreme environments, thanks to the sensitivity and selectivity of polymer-based detection systems. Moreover, sensory polymers have been integrated into process monitoring and safety measures in industrial applications.

Upon analysing the current landscape of sensory polymers and forecasting their trajectory, it becomes evident that their advancement has indeed spurred technological growth. However, their potential to significantly enhance people’s quality of life remains largely untapped. To address this, the challenge lies in seamlessly integrating them into everyday objects and systems, thereby making our surroundings safer and medical care more proactive. This requires meeting market requirements for their integration, catering to both specialized personnel and widespread use among the general population. Successfully tackling this multifaceted challenge is essential for unlocking the full potential of polymers, which has been a primary focus of sensor construction research since the early 21st century [86].

In addressing the complexities of polymer-based sensory materials, it is paramount to identify and understand the key challenges they present. These challenges encompass a wide spectrum of considerations, ranging from fundamental material properties to practical applications. Below, the main challenges currently faced by sensory polymers are summarized [1,3,13,66,86]:

- Enhancement of recognition mechanisms for rapid identification of target components, involving the exploration and selection of appropriate materials and the establishment of efficient signalling pathways;
- Optimization of the transduction process converting chemical interactions between receptors and targets into measurable properties (electrical, mechanical, or optical) to enhance detection capabilities;
- Reduction in detection or response time, crucial for identifying hazardous substances such as heavy metal cations or explosives;
- Development of solid-state pH and ion-selective sensors, providing more resilient and sturdy alternatives to existing technologies;
- Exploration of conducting polymer hydrogels for their practical application in biomedical sensors;
- Design and fabrication of novel sensor substrates and internal electrodes to accommodate new planar and solid-state sensor configurations;
- Advancement of novel biopolymers possessing sensory attributes, with a focus on broadening the spectrum of detectable target substances, particularly in biomedical settings, to augment disease detection and diagnosis capabilities;
- Enhancement of production methodologies to enable the commercialization of cost-effective, portable sensory devices suitable for non-specialized users, bridging the gap between fundamental research and real-world applications in response to societal needs;
- Automation of sensor manufacturing processes through the utilization of advanced fabrication methods like printing or semiconductor technologies, facilitating the production of compact sensor arrays;
- Improvement of signal processing technologies and instrumentation to enhance sensor performance and reliability.

## 6. Trends and Prospects

Taking into consideration the challenges previously discussed, along with the advancements in technology and their potential impact, in this section a condensed overview of the trends observed in sensory polymers will be provided. While detailed discussions on diverse polymer types and sensor categories have been presented in preceding sections, the focus here is on addressing challenges and limitations, offering a more general perspective on the prospects of polymer sensor development. We can conclude that the future trends in this field can be outlined as follows [1,3,13,66,86]:

- Multi-analyte sensing capabilities: advancements in sensor arrays enable the detection of multiple analytes, a crucial progression for complex applications like implantable sensors in medical diagnostics. These innovations highlight progress in partially selective sensing technologies;
- Improved molecular recognition: this trend focuses on refining the immobilization of receptor components via chemical modifications, with the goal of enhancing molecular recognition for superior selectivity. It also involves the utilization of novel materials for both transducers and chemical transduction strategies;
- Biochemical studies in non-aqueous media: exploring biochemical reactions in non-aqueous environments unveils novel biosensing prospects and broadens the scope of applications for polymer-based sensors beyond traditional settings;
- In-depth mechanism analysis: comprehensive analysis and further investigation into the specific reaction mechanisms of sensory polymers are imperative, as their complete understanding is lacking. This deeper exploration is crucial for the advancement of more sophisticated sensing technologies;
- Sensor miniaturization and integration: the development of compact sensor arrays with high-density, individually addressable elements facilitates advanced two-dimensional concentration mapping;
- Microtechniques and confined space phenomena: employing microtechniques and comprehending physicochemical phenomena in confined spaces is vital for adapting sensor devices to handle small sample volumes, particularly in biosensor research;
- Integration of artificial intelligence (AI): machine learning techniques can also optimize sensor designs and recognition algorithms, so the integration of AI holds immense potential for enhancing sensor performance and capabilities. AI algorithms can analyse vast amounts of data generated by sensor arrays, enabling real-time decision-making and adaptive sensing strategies.

## 7. Market

The transition from the laboratory to the successful commercialization of sensory polymers and seamless integration into everyday use represents the paramount challenge facing research efforts in this field. Overcoming this hurdle requires addressing various marketing challenges and ensuring that innovations are not confined to research environments. Instead, efforts must focus on facilitating their widespread adoption and practical implementation in real-world settings. This entails not only developing cutting-edge sensor technologies but also establishing effective strategies for market penetration and user acceptance.

In 2022, the global sensor market boasted an impressive value of USD 204.8 billion, with projections indicating a soaring trajectory to reach an estimated USD 508.64 billion by 2032. This surge represents a remarkable compound annual growth rate (CAGR) of 8.40% from 2023 to 2032, propelled by advancements across various sensor types. These include biosensors, optical, RFID, image, temperature, touch, flow, pressure, and level sensors, driven by established technologies such as the complementary metal–oxide–semiconductor (CMOS), micro-electromechanical systems (MEMS), and nanoelectromechanical systems (NEMS). With its potential for success, the sensory polymer sector stands at the forefront of this expansion, poised to make significant strides in vital industries like healthcare, automotive, industrial, IT/telecom, aerospace, and defence. The effective integration of sensory polymers into the market not only promises to enhance these sectors but also to usher in a new era of innovation and economic prosperity [87].

In an alternative market prospect, the industry reached a value exceeding USD 34 billion in 2020 and is forecasted to surge at a notable CAGR of 18% from 2021 to 2031. Featuring a wide range of sensor types, including flow, image, motion, pressure, temperature and humidity, touch, and water sensors, and driven by cutting-edge technologies like MEMS and CMOS, the market is on track to surpass a valuation of USD 208 billion by 2031. This upward trajectory underscores an industry in rapid evolution, diversifying its applications and leaving a significant mark across various end-use sectors. It signals a decade characterized by unprecedented growth and notable technological advancements [88].

Exploring a particular technology market prospect, such as the pH sensor market (comprising glass-type sensors, ion-sensitive field-effect transistor-ISFET sensors, among others), achieved a valuation of USD 603 million in 2022 and is forecasted to ascend to USD 1602 million by 2029 [89]. Another representative example is in the disease diagnostics area, where antibody-related tests, valued at USD 43 billion, command a substantial market share out of the total USD 112 billion market in 2021 [90]. The market for the advancement of novel biosensors reached approximately USD 27 billion in 2022 and is projected to surge to USD 50 billion by 2030. Hence, novel biosensors are considered indispensable assets in the burgeoning healthcare sector.

In this context, the implementation of new regulatory measures creates an opportunity by elevating the demand for materials and equipment tailored to monitor human-related risks, such as exposure to harmful vapours and gases in workplaces, water contamination from industrial waste, and the use of pesticides in agriculture. Polymers, renowned for their adaptability and capacity to be tailor-synthesized, have emerged as pivotal elements in the development of sensory devices for these essential areas [3].

Regarding the environmental sector, recent advancements have described the use of non-enzymatic optical and electrochemical biosensors for pesticide detection design from antibodies, aptamers, and MIPs, often combined with nanomaterials for improved performance. These biosensors, which include immunochromatographic and fluorescence assays, offer remarkable sensitivity and selectivity, making them well-suited for on-site applications due to their simplicity and visual detection capabilities. While electrochemical biosensors boast low detection limits, they tend to be more complex and less suitable for on-site use. Future enhancements in biosensor performance are anticipated to exploit novel nanomaterials, such as metal–organic frameworks and bimetallic nanoparticles, due to their catalytic and optical attributes, and aptamers and MIPs for their durability and cost-effectiveness. However, sustainability challenges, such as simplifying sample preparation and optimizing method reproducibility, must be tackled to facilitate the transition of these biosensors from the laboratory to the market.

On the other hand, the introduction of sensory polymers in the food sector offers significant benefits for both the food industry and consumers. These sensory polymers can lead to improved accuracy in quality control, waste minimization, heightened safety measures, and compliance with international food safety standards. For instance, in the market, sensors such as Timestrip, 3M’s MonitorMark, and Mitsubishi’s Ageless Eye indicate key environmental changes within food packaging, thus ensuring certain properties and characteristics of food quality [91]. Nonetheless, sensory systems in the food industry face competition from traditional analytical methods concerning cost, performance, and reliability. Moreover, in an industry where safety, quality control, and consumer trust are paramount, producers’ hesitance to embrace smart labels indicating deterioration levels arises from commercial apprehensions. Despite the crucial demand for innovative sensors capable of detecting freshness, spoilage, toxicity, and overall quality—potentially transforming the accuracy and reliability of food monitoring systems—commercial reservations impede their widespread adoption [38,92].

## 8. Conclusions

During this review, we have comprehensively examined and updated the challenges and emerging trends in the sensory polymer field. It becomes apparent that they hold significant untapped potential. This is primarily due to various challenges that must be addressed to enhance their technological maturity and facilitate their commercialization. The main obstacles include the necessity to improve the precision and sensitivity of sensory polymers and their capacity to detect a broader range of substances and conditions. Additionally, efforts are required to enhance the stability and durability of these polymers to ensure sustained functionality across diverse environments and applications. The successful commercialization of sensory polymers would benefit the scientific and technological community and yield positive impacts in daily life by providing innovative solutions for monitoring and controlling various processes and environments. Moreover, it could create new market opportunities for emerging enterprises, thereby stimulating economic growth and employment.
